# The effect of platelet-rich plasma on ferroptosis of nucleus pulposus cells induced by Erastin

**DOI:** 10.1016/j.bbrep.2024.101900

**Published:** 2024-12-24

**Authors:** Shi-lin Lian, Jie Huang, Yan Zhang, Yu Ding

**Affiliations:** a Orthopedics of TCM Senior Department, The Sixth Medical Center of PLA General Hospital, Beijing, 100048, China; b Department of Orthopedics, School of Medicine, South China University of Technology, Guangzhou, 510006, China

**Keywords:** Platelet-rich plasma, Intervertebral disc degeneration, Ferroptosis, Nucleus pulposus cell, Mitochondria

## Abstract

**Background:**

Intervertebral disc degeneration (IVDD) has been linked to ferroptosis, a type of programmed cell death. The role of platelet-rich plasma (PRP) in mitigating ferroptosis in nucleus pulposus (NP) cells within IVDD remains unclear.

**Purpose:**

This study aims to verify the effectiveness of PRP in reducing ferroptosis in NP cells induced by Erastin.

**Methods:**

Primary NP cells were isolated from SD rats, and a ferroptosis model was established using Erastin. PRP was prepared and applied to assess its impact on ferroptosis-related markers, including reactive oxygen species (ROS), iron content, and glutathione peroxidase 4 (GPX4). The effects of PRP on the ultrastructure of NP cells were also observed using transmission electron microscopy (TEM).

**Results:**

PRP treatment significantly restored GPX4 levels (431.47 ± 4.70 ng/L vs. 69.70 ± 4.06 ng/L, P < 0.05), reduced ROS levels (45.06 ± 3.78 vs. 155.36 ± 3.56, P < 0.05), and decreased iron content (32.24 ± 096 μg/L vs. 59.25 ± 3.72 μg/L, P < 0.05) in ferroptotic NP cells compared to the sham group. Additionally, PRP significantly increased the expression levels of collagen Ⅱ (0.72 ± 0.02 vs. 0.33 ± 0.02, P < 0.05) and aggrecan (0.81 ± 0.01 vs. 0.31 ± 0.02, P < 0.05) compared to the sham group. TEM analysis also showed improvements in mitochondrial ultrastructure. These findings suggest that PRP can alleviate ferroptosis and promote cellular recovery.

**Conclusions:**

The study demonstrates the potential of PRP as a therapeutic intervention in IVDD by mitigating ferroptosis in NP cells, offering a new theoretical basis for PRP treatment in degenerative disc diseases.

## Highlights

• This is the first study about PRP on ferroptotic NP cells regarding IVDD.
• PRP can restore the ultrastructure of mitochondria of ferroptotic NP cells.
• The content of ROS, Fe, GPX4, and GSH-PX enzyme activity can be restored by PRP.
• The content of ECM produced by NP cells can also be restored in ferroptosis model.
• This study provides a new insight for PRP treatment in IVDD.

## 1 Introduction

The incidence of low back pain (LBP) is extremely high, and the mechanisms of LBP are correlated with muscular [[1], [2], [3]], spinal [[4], [5], [6]], neurogenic factors [7,8], etc. Among them, intervertebral disc degeneration (IVDD) is the most studied one [[9], [10], [11]]. The intervertebral disc, composed of the cartilage endplate, annulus fibrosus (AF), and nucleus pulposus (NP), is a fibrocartilage connecting structure between the adjacent vertebral bodies [12]. Intervertebral disc is the largest avascular tissue in human body, and its nutrient supply mainly depends on the infiltration of cartilage endplate, which leads to the difficulty of intervertebral disc repairing under the circumstances of IVDD. When the intervertebral disc undergoes degeneration, the load-bearing capacity of the disc decreases, and the biomechanical stability of the entire spine also deteriorates [13]. The manifestation of IVDD includes the decrease of water content of NP tissue, bulge or protrusion or extrusion of NP tissue, generation of blood vessel at the margin of AF [14,15], rupture of the AF, etc. The degeneration of NP tissue is considered to be the initiating factor of IVDD [16,17]. Currently used surgical techniques for IVDD associated diseases, including open surgery and endoscopic surgery, require the removal of herniated disc tissue, which is mainly NP tissue, to achieve the goal of relieving compression and alleviating clinical symptoms. However, due to the removal of tissue resulting in decrease in the amount of active NP cells and extracellular matrix (ECM), the alterations caused by degeneration have not been fundamentally improved.

Platelet-rich plasma (PRP) is a plasma portion containing high concentration of platelets obtained by centrifugation from peripheral blood. There are abundant of growth factors and other components within platelets. These active components offer the ability of PRP to promote cell proliferation and ECM synthesis, and alleviate the inflammatory response. The main factors in PRP include [18] transforming growth factor (TGF), platelet-derived growth factor (PDGF), vascular endothelial growth factor (VEGF), insulin-like growth factor (IGF), epidermal growth factor (EGF) and fibroblast growth factor (FGF). After the activation of PRP, the alpha particles in platelets can release these growth factors contained, which can promote the repairing of tissue synergistically. Therefore, compared with the application of one certain growth factor, PRP can achieve a better therapeutic effect. The application of PRP to the intervertebral disc tissue after herniation removal can delay the degeneration process and promote tissue repair.

In 2012, the team of Brent R. Stockwell [19] proposed a new programmed cell death (PCD) named ferroptosis. The morphology of ferroptotic cells is different from apoptotic cells which are characterized by shrinkage, nucleus condensation, and apoptotic body formation. Typical features of ferroptosis includes the shrinkage of mitochondria volume, reduction of cristae number and vague cristae [20]. Reports about the correlation between ferroptosis and IVDD have suggested that ferroptosis may be one of the important mechanisms leading to the occurrence and development of IVDD [[21], [22], [23]]. Studies have shown that PRP has a positive effect on ferroptosis-related diseases including endometrial epithelium regeneration, sepsis-induced myocardial dysfunction, (SIMD), Parkinson's and amyotrophic lateral sclerosis, etc. The morphological changes of ferroptotic cells and the related markers of signaling pathways can be intervened by PRP. Whether the effect of PRP on IVDD is correlated with ferroptosis is still unknown.

Therefore, in order to explore whether ferroptosis in IVDD can be mitigated by the application of PRP, ferroptosis model of NP cells was established by Erastin, confirmed by TUNEL and transmission electron microscope (TEM), and then model NP cells were intervened by PRP to verify whether the ferroptotic NP cells could be mitigated by PRP.

## 2 Materials and methods

The experimental animals were 6 male SD rats, 200g each, SPF. The animals were bought from Beijing HFD Bio-technology Co., Ltd. The experiment protocol was approved by the Laboratory Animal Ethics Committee of Kangtai Medical Laboratory Service Hebei Co., Ltd. (MDL2022-10-12-01). All animal experiments were carried out in accordance with the U.K Animals (Scientific Procedures) Act, 1986 and associated guidelines.

### 2.1 Overall design

NP cells were isolated and cultured from rat NP tissue. Besides, PRP was prepared using peripheral blood and was detected concentration of corresponding cytokines. After that, the cultured NP cells were added with 10 μmol Erastin and were incubated for 24 h to induce ferroptosis model in which the cells were divided to model group (treated by Erastin) and control group (treated by PBS). The control group was treated with an equivalent volume of PBS throughout the experiment to exclude the influence of other variables, ensuring that the observed effects were solely due to ferroptosis induced by Erastin. NP cells in model group and control group were analyzed through TUNEL assay. Furthermore, the ultrastructure of cells in the two groups was monitored by TEM. The ferroptosis model cells were then subdivided into PRP intervention group and sham group before executing latter intervention and experiments. Additionally, in the PRP intervention experiments, the PRP intervention group was treated with 10 % PRP lysate, while the control (sham) group was treated with an equivalent volume of PBS. These control groups were set up to verify the specific effects of PRP intervention on NP cells. Ferroptosis-related markers, which includes reactive oxygen species (ROS) content, Fe content, glutathione peroxidase (GSH-PX) activity, and glutathione peroxidase 4 (GPX4) content, along with ECM protein were detected. Furthermore, NP cells and mitochondria morphology were monitored by TUNEL and TEM respectively to determine the effect of PRP intervention.

### 2.2 PRP preparation

The blood was collected from abdominal aorta of SD rats, using vacuum tubes with ACD-A solution to extract 5∼8 ml of blood for each rat. The whole blood and ACD-A were blended in a ratio of 9:1. The preparation procedure of PRP was as follows. The initial centrifugation was performed at 1000×*g* relative centrifugal force (RCF) for 15min. Subsequently, the diluted RBC layer and the upper portion were transferred into a new centrifuge tube. Then, a second centrifugation at 300×*g* RCF for 5min was conducted. The supernatant fluid above the RBC layer was aspirated and transferred to another centrifuge tube. Finally, a centrifugation at 1200 rpm for 10min was executed. The pellet at the bottom was retained to obtain platelet-rich plasma (PRP) after resuspension. Excess supernatant fluid was removed, resulting in approximately 1/10 volume of PRP comparing to the original whole blood volume.

PRP and whole blood were activated by the activator which was prepared by mixing 1000U of porcine thrombin lyophilized powder with 5 ml of calcium chloride solution. After adding the activator, the centrifuge tubes containing the PRP samples were transferred to a 37 °C water bath for 30min. Subsequently, the tubes were removed from the water bath and centrifuged at 3000×*g* RCF for 20min. The supernatant fluid was then extracted and obtained the PRP lysate. The whole blood lysate was obtained according to the same protocol as PRP mentioned above.

### 2.3 ELISA assay

The concentrations of target cytokines of prepared PRP lysate and lysate from normal whole blood were compared using ELISA assays. For each cytokine, 5 replicates were established for testing, with 7 wells designated for standard samples. The cytokines tested included PDGF-BB, TGF-β1, IL-1β, and TNF-α. The procedure was performed according to the instructions provided in the assay kit. The results were then measured by determining the absorbance (A) at 450 nm using an enzyme-linked immunosorbent assay (ELISA) reader. Standard curves were generated based on the data from different sample groups, and the concentrations of the respective factors in the samples were calculated accordingly.

### 2.4 Isolation and cultivation of NP cells

SD rats were euthanized through cervical dislocation method. The rats were immersed in 75 % ethanol for 5min before further processing. Under aseptic conditions, the thoracolumbar spine of the rat was dissected, and the adjacent muscles and tissues were carefully separated to expose the intervertebral disc. The annulus fibrosus was split by sterile scalpel, and the gelatinous NP tissue was extracted. After repeated rinsing with PBS solution, the NP tissue was finely minced by ophthalmic scissors to facilitate tissue digestion. The minced tissue was then transferred to a 15 ml centrifuge tube and digested in DMEM-F12 (1:1) culture medium containing 10 % type Ⅱ collagenase (37 °C, 5 % CO_2_, 4 h). Once the digestion process was completed, complete culture medium was added to terminate the digestion. The mixture was gently pipetted to create a cell suspension. The centrifuge tube was then placed in a low-speed centrifuge at 1000 rpm for 10min. Afterward, the supernatant fluid was discarded, and the pellet was transferred as much as possible to a 25 cm^2^ culture flask. The flask was placed in a 37 °C, 5 % CO_2_, saturated humidity incubator for cultivation. Once cells migrated out, excess tissue fragments were removed. Medium renewal was performed every 3d.

### 2.5 Ferroptosis model of NP cells

Erastin is a cystine/glutamate transporter (System Xc^−^) inhibitor that can suppress reduced glutathione (GSH) synthesis, reduce the concentration of the substrate for GPX4 reactions, and can lead to an increase of intracellular ROS levels, thereby inducing ferroptosis.

Stable-growing NP cells were seeded onto a 96-well plate (1000 cells/100 μl) and divided equally into the model group and the control group. Model group: 10 μmol/L Erastin was added to the wells and incubated for 24 h. Control group: an equal amount of PBS solution was added for control.

### 2.6 Intervention of cells

The NP cells of model group were cultured in a 96-well plate and subdivided evenly into the PRP intervention group and sham group. In the PRP intervention group, intervention was conducted using 10 % PRP lysate. While in the sham group, an equivalent volume of PBS solution was applied. 3 replicates were set for each group. The intervention spanned 1 week, involving media renewal every 2d. Following media renewal, the same volume of PRP and PBS solution were added.

### 2.7 TUENL assay

The cell death and DNA damage in ferroptotic cells can be detected by TUNEL assay. The principle involves the utilization of the 3′-OH exposed by DNA fragmentation. These ends are then labeled with a red fluorescence probe, Cy3 (cyanine 3)-labeled dUTP, through the catalytic action of terminal deoxynucleotidyl transferase (TdT). This labeling enables detection through fluorescence microscopy or flow cytometry.

In the TUNEL assay, the comparison between the NP cells from the model group (Erastin-induced group) NP cells and control group NP cells (PBS treated) was conducted initially. Subsequently, the cells in model group were subjected to PRP intervention and were compared with cells of sham group subjected to PBS solution by TUNEL assay. 4 % paraformaldehyde were added to each slide to fix the cells under room temperature for 10min. 3 % hydrogen peroxide was added to the cells in both groups. Incubation was carried out at room temperature for 10min. 100 μl of enhanced immunostaining permeabilization buffer was added to the glass slides and incubated in a humid chamber for 5min. 50 μl TUNEL detection solution was added to the two groups of cells and incubated in the dark for 60min. The slides were sealed with antifade mounting medium and observed under a fluorescence microscope.

### 2.8 TEM

The ultrastructure of NP cells, especially the morphology of mitochondria, was observed by TEM, discerning alterations in cellular ultrastructure after ferroptosis induction and subsequent PRP intervention. In cells undergoing ferroptosis, mitochondria display shrinkage, diminished cristae, cell membrane rupture, and vesiculation. These subtle structural modifications can be distinctly observed through the application of TEM. TEM was conducted after the ferroptosis model established which include the model group cells and the control group cells and was conducted again to observe the alterations in PRP intervention group and sham group later.

After centrifugation, the cells were fixed with 2.5 % glutaraldehyde solution for 2–4 h. Low melting point agarose was added to the cell suspension. 2.5 % glutaraldehyde solution was used to fix the cell pieces. Samples were rinsed 3 times with phosphoric acid rinse solution for 15min each, and were then placed in 1 % osmium acid and fixed at 4 °C for 2 h. After that, the samples were dehydrated (30%-50%-70%-80%-95%-100%-100 %) by alcohol gradient dehydration method for 5min each time. Osmotic embedding was carried out according to the ratio of propylene oxide: embedding solution = 2:1 for 2 h; Propylene oxide: embedding solution = 1:2 for 3 h; Pure embedding solution, room temperature, overnight; Pure embedding solution, room temperature, 3–4 h. The embedding plate was placed in a 60 °C oven for 48 h, and the embedding block was removed after the resin polymerization was complete. The resin block is cut into 70 nm thin slices with an ultra-thin microtome and fished out with copper mesh. The slices were stained with uranium acetate saturated alcohol solution and 2.7 % lead citrate solution for 8min chronologically.

### 2.9 Biochemical assay

The ferroptosis-related markers in PRP intervention group cells, sham group cells and normal cells were compared by biochemical assay. The biochemical assay included quantification of intracellular ROS levels, iron (Fe) content, GSH-PX enzyme activity, and GPX4 levels. By contrasting normal cells group, model group and PRP intervention group, the alterations in ferroptosis-related biochemical markers were observed.

### 2.10 GPX4 content

The samples were diluted by adding the standard dilution to 4000 ng/L, 2000 ng/L, 1000 ng/L, 500 ng/L, and 250 ng/L. Then the antigen working solution were prepared by mixing the biotin antigen dilution and the concentrated biotin antigen. Avidin HRP was transferred to the dilution bottle and mixed to obtain the avidin HRP working solution. The cell suspension was diluted using PBS and adjusted to 10^6^/ml. To release the intracellular components, the cell suspension underwent repeated freeze-thaw and centrifugation of 2000 rpm for 20 min. The supernatant fluid was collected. The further detailed operation was performed according to the instructions of the kit.

### 2.11 GSH-PX enzyme activity

GSH-PX catalyzes the reaction of hydrogen peroxide with GSH in the form of H_2_O and oxidized glutathione (GSSG). By measuring the consumption of the reactant GSH in this enzymatic reaction, the activity of the enzyme can be calculated. The cell supernatant fluid was extracted using centrifugation at 3000 rpm for 10min. The application of reagents was conducted according to the instructions of the kit in Table 1. The mixed components were centrifugated at 4000 rpm for 10min. 1 ml of supernatant fluid was extracted for color reaction which was performed by measuring the OD value (412 nm) of each tube. Calculation of the enzyme activity was conducted by the instructions of the kit.

**Table 1:** The details of the reagents applied in GSH-PX kit.

	Enzyme Tube	Non-enzyme Tube
1 mmol/L GSH(ml)	0.2	0.2
Sample Volume(ml)		0.2
Water Bath, 37 °C, 5min		
Reagent 1(ml)	0.1	0.1
Water Bath, 37 °C, 5min		
Reagent 2(ml)	2	2
Sample Volume(ml)	0.2	

### 2.12 Fe content

1 ml of extract solution was added to 5 × 10^6^ cells. The cells were then sonicated in an ice water bath for 3s, 30 times. Then the cells were centrifuged at 12,000 rpm for 10min under 4 °C, and the supernatant fluid was placed on ice to be tested. The microplate reader was pre-warmed for 30 min, and the wavelength was set to 562 nm. The content of Fe with cells was calculated based on the absorbance 562 nm. The calculation was conducted according to the equation from the instructions of the kit.

### 2.13 ROS content

The cell suspension was centrifuged at 1000 rpm for 5min. The serum-free medium was added to resuspend cells, followed by probe addition at an initial concentration of 10 μM. Cells added with culture medium alone served as a negative control, and another portion of cells added with probe and ROS hydrogen donor served as a positive control. Cells were incubated at 37 °C for 40min. The optimum excitation wavelength was set at 488 nm, and the optimum emission wavelength was set at 525 nm.

### 2.14 Quantitative real-time fluorescence polymerase chain reaction analysis

The RNA transcription of FPN, collagen Ⅱ, and aggrecan was compared among normal cells, PRP intervention group and sham group. TRIzol was added to cell sample for lysis. 700 μl of isopropanol was added to the supernatant obtained after centrifugation and mixed thoroughly. 75 % ethanol solution was used to wash the sediment, followed by air-drying at room temperature. The sediment was dissolved using 50 μl DEPC. Measurement was performed using a nucleic acid concentration detector. Using the Superscript Ⅲ reverse transcription kit, Reaction System 1 was established. After thorough mixing, the system was centrifuged at 65 °C for 5min, and then transferred to an ice bath to create Reaction System 2. The system was immersed in a 42 °C water bath for 60min. Following this, it was transferred to a system at 85 °C for a 10min reaction to inactivate the reverse transcriptase enzyme. The product was stored at −20 °C for subsequent procedures. The primers designed were elaborated in Table 2.

**Table 2:** Primer design information.

Target Name	Primer	
actin	F	CTGAACGTGAAATTGTCCGAGA
	R	TTGCCAATGGTGATGACCTG
Collagen Ⅱ	F	CGCCATGAAAGTCTTCTGCAACA
	R	CACCAGTTCTTCCGAGGCACA
Aggrecan	F	CGCTGGTCTGATGGACACTC
	R	AGATCATCACTACGCAGTCCT
FPN	F	GGAGCATCAGCAATAACTGGA
	R	AACAAGGCCACATTTTCGAC

### 2.15 Western blot analysis

The cells were washed with PBS solution, then RIPA buffer was used for lysis of NP cells. After extraction of total proteins, bicinchoninic acid (BCA; MDL, MD913053, China) working solution was prepared to analyze the concentration. The electrophoresis of the sample protein was conducted by using the sodium dodecyl sulfate polyacrylamide gel electrophoresis (SDS-PAGE; Bio-Rad, USA), and the transfer operation was conducted by using the polyvinylidene fluoride (PVDF; Millipore, Billerica, USA)membrane. The block of membrane was performed by using 5 % skimmed milk under room temperature for about 1 h. Then primary antibody of collagen Ⅱ (1:1000; AF0135, Affinity Biosciences), aggrecan (1:1000; DF7561, Affinity Biosciences) and actin (1:1000; #AF7018, Affinity Biosciences) were applied for reaction at 4 °C overnight. After reaction, the membrane was washed with 1 × TBST for 10min, three times. HRP goat anti-rabbit IgG (1:1000; 511103; Zen Bio) was used to incubate the membrane.

### 2.16 Statistical analysis

Statistical analysis and graph plotting were conducted using GraphPad Prism 8 (GraphPad Software Inc., La Jolla, USA). Data are presented as mean ± standard deviation ($\bar{x}$ ± SD), with 95 % confidence intervals calculated. For comparisons between two groups, Student's t-test was used, assuming normal distribution and equal variances. For comparisons involving three or more groups, one-way analysis of variance (One-way ANOVA) was conducted, followed by post hoc analysis to assess significance between groups. The choice of these statistical tests is based on their reliability under conditions of small sample sizes and homogeneity of variances. Significance was set at α = 0.05, with statistical significance considered when *P* < 0.05.

## 3 Results

### 3.1 ELISA assay

**Fig. 1:**
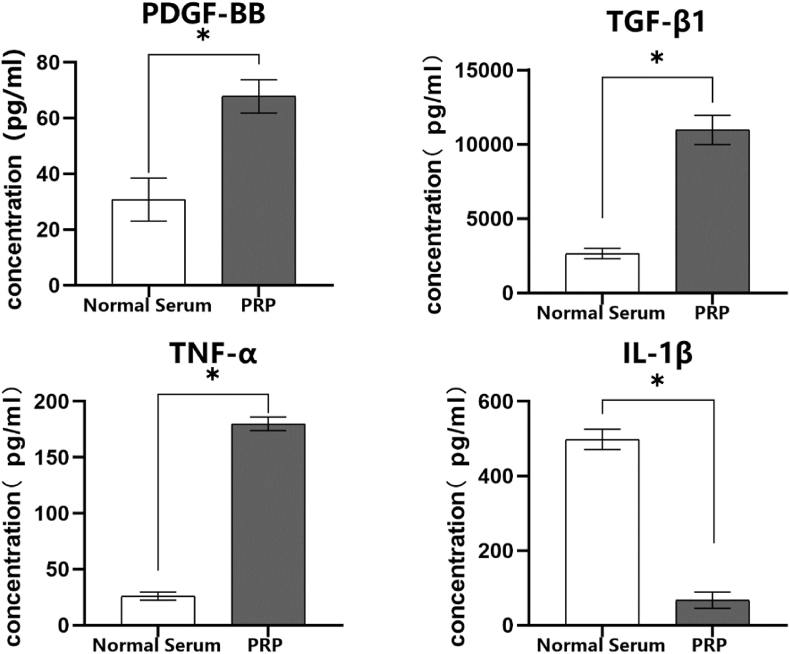
Concentration of growth factors and proinflammatory cytokines within normal serum and PRP lysate (ns means *P>*0.05, ∗ means *P<*0.05).

The concentration of the cytokines in PRP sample was detected by the ELISA kit (Fig. 1). The concentration of PDGF-BB, TGF-β, TNF-α, IL-1β in normal serum was as follows respectively: 30.71 ± 7.70 pg/ml, 2645.20 ± 354.21 pg/ml, 25.89 ± 3.63 pg/ml, 498.14 ± 27.35 pg/ml. These results of PRP sample were: 67.81 ± 6.00 pg/ml, 10966.80 ± 980.36 pg/ml, 180.00 ± 6.21 pg/ml, 67.29 ± 21.56 pg/ml. The difference in these results between the two groups was statistically significant (*P* < 0.05).

### 3.2 TUNEL and TEM results for Erastin-induced ferroptosis model of NP cells

**Fig. 2:**
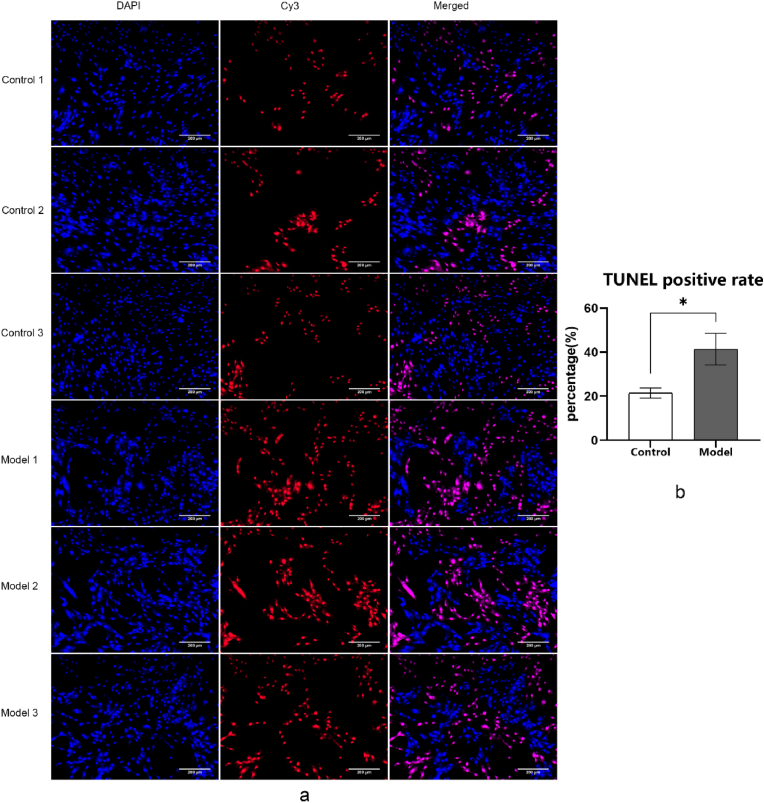
TUNEL analysis of control group and model group after Erastin modeling (DAPI marked normal cells, and Cy3 indicated TUNEL positive cells, which showed a significant difference in TUNEL positive rate between the two groups, and a significantly higher proportion of positive rate in the model group, scale bar = 200 μm).

The death of NP cells can be positively stained as red in TUNEL assay. As shown in the results (Fig. 2), the TUNEL positive rate in the control group was significantly lower compared to the model group. Through quantitative analysis, TUNEL positive rate in the control group was 21.41 ± 2.34 %, while the result in model group was 41.29 ± 7.22 %. The difference between these two results were statistically significant(P = 0.014). This indicated that the ferroptosis-induced cell model of NP cells treated with 10 μmol/L Erastin for 24 h exhibited a significantly higher TUNEL positive rate compared to normal NP cells without Erastin intervention, which meant the success of the establishment of the ferroptosis model of NP cells.

Furthermore, Ferroptosis is a form of programmed cell death (PCD) distinct from apoptosis, autophagy, or necroptosis. Typical features of regular apoptosis include cell shrinkage, chromatin condensation, DNA degradation and fragmentation, membrane blebbing, and the formation of apoptotic bodies. In contrast, cell morphology during ferroptosis differs from apoptosis or necrosis and involves specific organelles, including mitochondria, endoplasmic reticulum, and lysosomes. Among these organelles, mitochondrial changes are particularly prominent, characterized by reduced volume, decreased cristae density, concomitant loss of membrane potential, and increased permeability. These alterations can be clearly observed under TEM.

**Fig. 3:**
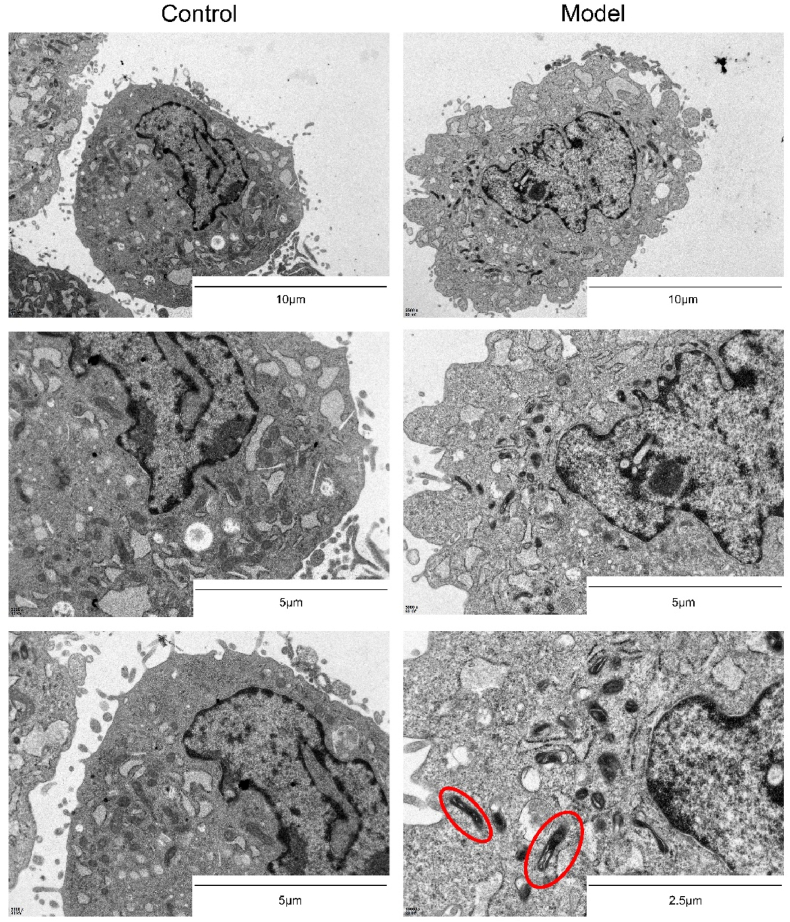
TEM showed the cellular microstructure of the control group versus the model group after Erastin modeling (The structures circled by the red ovals are mitochondria. Significant changes in mitochondria could be seen in the figure: shrinkage, density increase, decreased mitochondrial cristae).

TEM results (Fig. 3) revealed that, in the normal control group, normal morphological structures without specific changes associated with ferroptosis were identified in NP cells. Mitochondria was also intact with clear cristae. Conversely, typical morphological signs of ferroptosis could be perceived in Erastin-induced ferroptosis NP cells in model group, including membrane blebbing, disrupted internal structures, and pronounced mitochondrial alterations. Mitochondria was confirmed to appear increased density, reduced volume, and a noticeable decrease or loss of cristae, indicating severe mitochondrial damage. The occurrence of ferroptosis induced by Erastin was verified by these significant changes in mitochondria.

### 3.3 Biochemical assay

GPX4, a central focus of ferroptosis research, plays a vital role in maintaining cellular homeostasis by reducing polyunsaturated fatty acid peroxides to non-toxic lipids in coordination with GSH. During the occurrence of ferroptosis, there are notable alterations in the content or activity of GPX4. Additionally, the levels of ROS and Fe also correspondingly change due to the onset of ferroptosis.

**Fig. 4:**
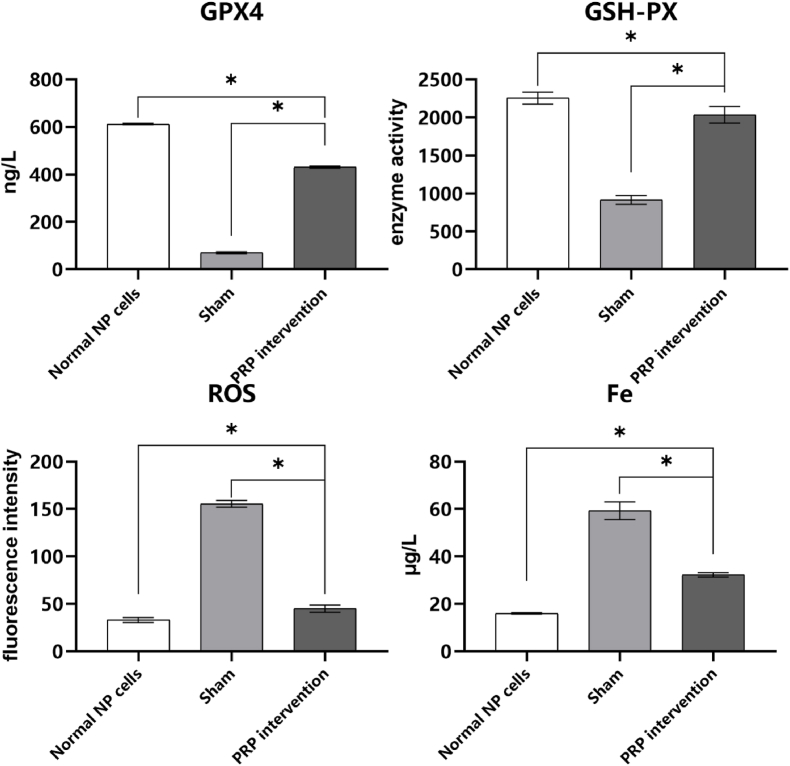
Results of GPX-4, GSH-PX, Fe, ROS determined in normal NP cells, sham group and PRP intervention group (ns means *P* > 0.05, ∗ means *P* < 0.05).

Results revealed that (Fig. 4) following Erastin-induced modeling, significant molecular alterations were observed in NP cells of the model group compared to normal NP cells. Specifically, GPX4 enzyme levels significantly decreased, GSH-PX enzyme activity markedly declined, and there was a substantial increase in relative ROS levels and Fe accumulation. Upon PRP treatment of model cells, these changes were notably reversed. GPX4 enzyme levels (ng/L) were as follows: normal cells 612.49 ± 3.10, sham group 69.70 ± 4.06, PRP intervention 431.47 ± 4.70. GSH-PX (enzyme activity unit) values: normal cells 2254.21 ± 79.10, sham group 914.02 ± 56.91, PRP intervention 2035.514 ± 109.17. Iron levels (μg/L): normal cells 16.01 ± 0.03, sham group 59.25 ± 3.72, PRP intervention 32.24 ± 0.96. ROS fluorescence intensity values: normal cells 33.12 ± 2.68, sham group 155.36 ± 3.56, PRP intervention 45.06 ± 3.78. All differences were statistically significant (*P* < 0.05).

### 3.4 Quantitative real-time fluorescence polymerase chain reaction analysis

**Fig. 5:**
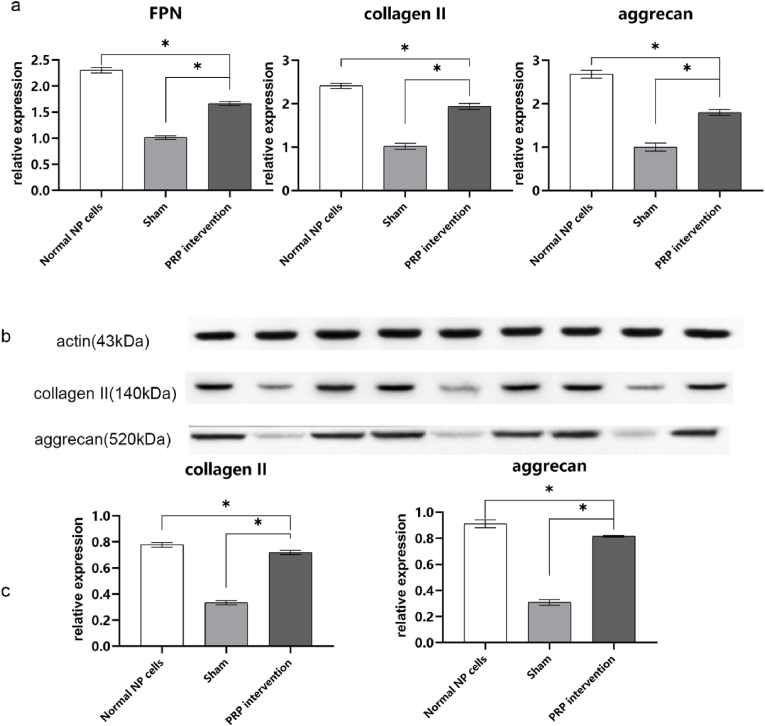
a: column of PCR test of FPN, collagen Ⅱ, aggrecan RNA expression results; b: primitive strips of Western blot (actin, aggrecan, collagen Ⅱ); c: comparison of expression of collagen Ⅱ and aggrecan after PRP intervention of ferroptosis model NP cells (ns means *P* > 0.05, ∗ means *P* < 0.05).

The results (Fig. 5, a) of RNA relative expression levels of FPN were 2.30 ± 0.05 in normal cells, 1.01 ± 0.04 in sham group, 1.66 ± 0.04 in PRP intervention group. And these results of collagen Ⅱ were 2.41 ± 0.06 in normal cells, 1.01 ± 0.07 in sham group, 1.93 ± 0.07 in PRP intervention group. The results of aggrecan were 2.68 ± 0.09 in normal cells, 1.00 ± 0.09 in sham group, and 1.80 ± 0.07 in PRP intervention group. The differences were significant (*P* < 0.05).

From the results, the RNA relative expression of the three functional proteins was significantly reduced from the comparison of the normal cells and the sham group. And the RNA relative expression of the three functional proteins after the intervention of PRP was obviously restored, which was closer to the expression of normal cells. However, statistically speaking, the difference in the RNA relative expression between the PRP intervention group and the normal cells was still significant (*P* < 0.05). The RNA relative expressions of FPN, collagen Ⅱ, and aggrecan of normal cells was still higher than those of ferroptosis model cells accepted PRP intervention which labeled as PRP intervention group.

### 3.5 Western blot analysis

The results indicated (Fig. 5b–c) that the relative expression levels of collagen Ⅱ (actin as a benchmark) in the three groups were as follows: normal cells 0.77 ± 0.02, sham group 0.33 ± 0.02, PRP intervention group 0.72 ± 0.02. The relative expression levels of aggrecan (actin as a benchmark) in the three groups were as follows: normal cells 0.91 ± 0.03, sham group 0.31 ± 0.02, PRP intervention group 0.81 ± 0.01. All differences were statistically significant (*P* < 0.05). These results indicated that in normal cells, the relative expression levels of the ECM main components, collagen Ⅱ and aggrecan proteins, were significantly higher than those in PRP intervention group and sham group. Additionally, after PRP intervention, the relative protein expression levels in the NP cells of the ferroptosis model cells significantly increased, approaching levels seen in normal cells. However, statistically significant differences in relative expression levels persisted between the PRP intervention group and normal cells.

### 3.6 TUNEL assay

**Fig. 6:**
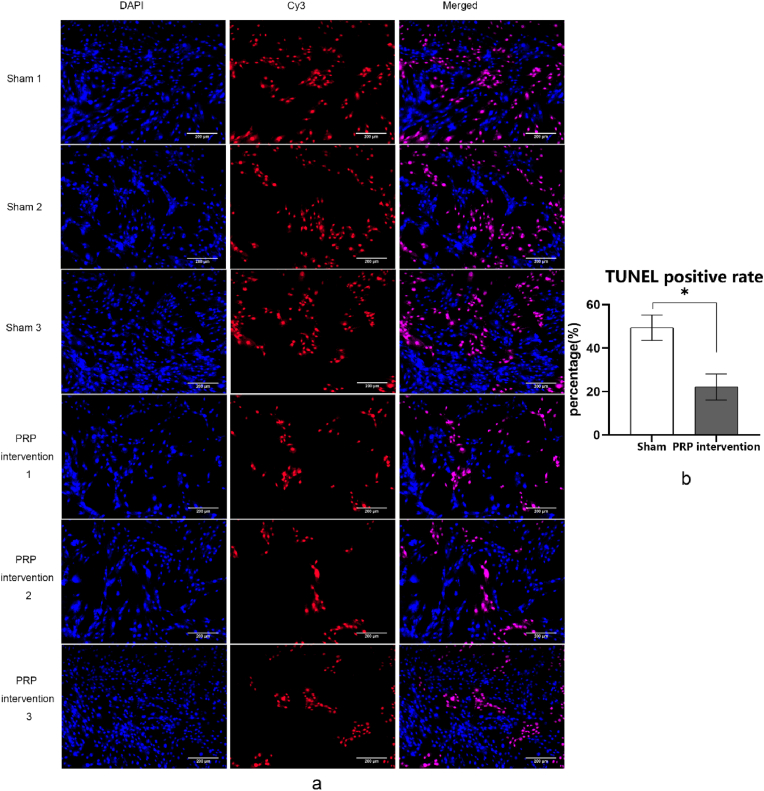
TUNEL analysis of NP cells intervened by PRP and PBS after ferroptosis modeling (results showed a significant difference in TUNEL positive rate between the two groups, and the proportion of positive cells in the PRP intervention group decreased significantly after PRP intervention, scale bar = 200 μm).

As shown in the results of TUNEL after intervention (Fig. 6), the rate of TUNEL positive cells in the NP cells of PRP intervention group was significantly reduced, and the density of dead cells was significantly reduced compared with the NP cells in the sham group treated with PBS. The TUNEL positive rate was quantitatively calculated, and the result of TUNEL positive rate of NP cells in the sham group and PRP intervention group was 49.39 ± 5.80 % and 22.12 ± 6.03 % respectively. There was a significant difference between the two results (*P* < 0.05). This revealed that TUNEL positive rate of cells was significantly reduced after PRP intervention, and PRP could reduce the cell death rate of ferroptosis model cells.

### 3.7 TEM

**Fig. 7:**
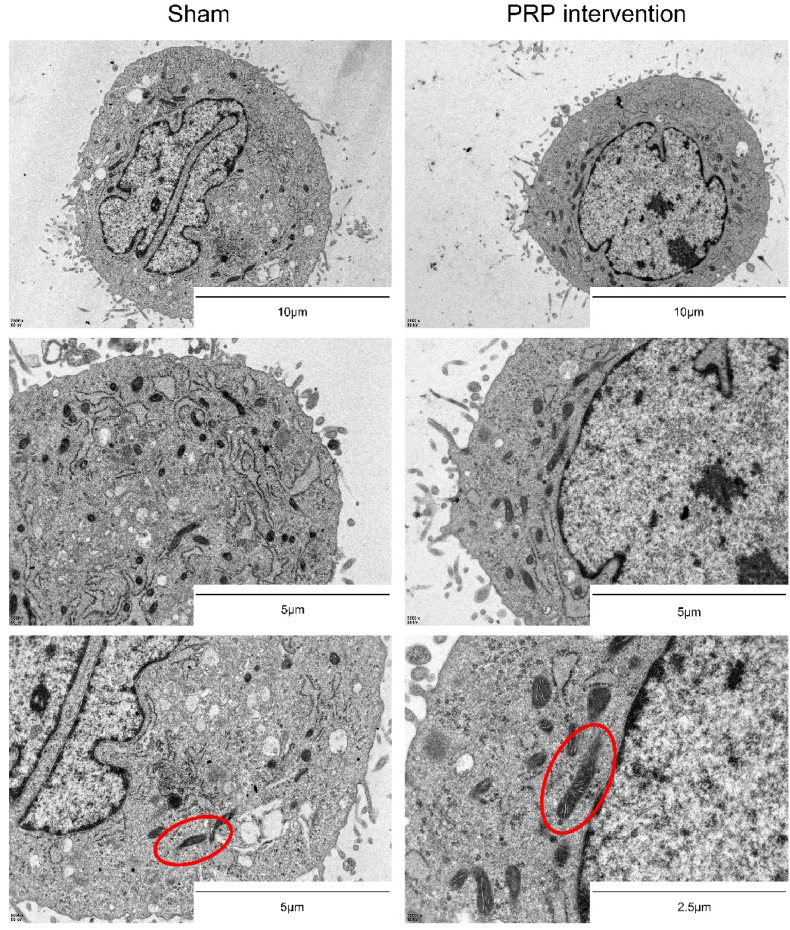
TEM results showed the ultrastructure of ferroptotic NP cells after PRP intervention (The structures circled by the red ovals are mitochondria. Mitochondrial morphology was significantly improved after PRP intervention.).

As shown in Fig. 7, mitochondria of NP cells in the sham group showed typical signs of ferroptosis model cells: decreased mitochondrial volume, increased mitochondrial density, and disappeared mitochondrial cristae. In contrast, NP cells in the PRP intervention group had smaller mitochondrial density, larger mitochondrial volume, and mitochondrial cristae than those in the sham group. These results suggest that the ultrastructure of NP cells can obtain restoration after the intervention of PRP compared to the sham group cells which were treated with PBS solution.

## 4 Discussion

From the results, the concentrations of growth factors of PRP, which include the PDGF-BB, TGF-β1, were significantly higher than those of normal serum. The concentration of IL-1β was much lower than that of normal serum. While the concentration of TNF-α was much higher than that of normal serum. These results indicated that PRP preparation was successful. The TUNEL positive cells in ferroptosis model group was significantly higher than control group confirmed by the quantitative analysis. According to the TEM results of model group and control group, the morphology alterations of NP cells in model group induced by Erastin reagent was verified to have membrane disruption and mitochondrial damage. Although, the results above revealed that the positive effects of PRP cannot fully restore the morphology and function of ferroptotic NP cells, of which the differences were statistically significant compared with normal NP cells. Still, the positive effect of PRP on ferroptotic NP cells was authenticated to improve the morphology and function of NP cells, among which the restoration of mitochondria morphology was the most significant improvement. The morphology of mitochondria revealed that the oxidative stress was alleviated because of the application of PRP. Furthermore, the results of ferroptosis-related markers have also been significantly improved including GPX4, GSH-PX, ROS, Fe. In addition, PRP can also promote the recovery of basic cellular functions in ferroptotic NP cells. The ECM synthesis function, which includes collagen Ⅱ and aggrecan, has been restored compared with ferroptosis model cells after PRP intervention. The differences of FPN in PCR suggested that the Fe overload state can be mitigated by the intervention of PRP, which was further confirmed by the biochemistry analysis.

PRP is widely used in multidisciplinary fields because of its satisfying function of promoting tissue, cell regeneration, besides the convenience of access. In orthopaedics surgery and sports medicine, PRP is applied for the treatment of fractures [24], osteoarthritis [25], tendon [26] or ligament repairing [27], and for reduction of the posttraumatic recovery time, because its promotion of the proliferation and differentiation of chondrocytes, acceleration of bone growth, reduction of joint pain, and alleviation of inflammations. Multiple growth factors can be released from activated PRP and applied to the target spot. The administration of PRP in the treatment of IVDD can gain satisfactory results. Numerous reports have confirmed the effect of PRP in animal IVDD models. Gelalis [28] et al. used 18 female New Zealand rabbits to establish IVDD models through needle puncture method, and animals were divided into two groups. One group received PRP injections, while the other group received saline injections. The experimental results revealed a significant reduction in the severity of IVDD in the PRP group compared to the control group which received saline injections. Moreover, obvious manifestations of tissue regeneration and repairing of IVD tissue were observed in PRP group. The degree of degeneration was assessed through HE tissues grading and expression differences of Collagen II. This animal experiment suggested that in the early stages of IVDD, PRP therapy can slow down the degeneration process and promote tissue regeneration and repairing. Xu [29] et al. established in vivo and in vitro mouse models of IVDD using puncture and hydrogen peroxide exposure methods. They confirmed the occurrence of NOD-like receptor thermal protein domain associated protein 3 (NLRP3)-mediated cell pyroptosis, apoptosis, and inflammatory responses during the progression of IVDD. However, the adverse effects on NP cells in the model were reversed by the PRP-derived exosomes which were gained by the isolation and purification. This suggests that exosome miR-141-3p from PRP may delay the progression of IVDD by inhibiting the NLRP3 inflammasome-induced inflammatory response. Furthermore, Yang [30] and his colleagues found the changes in NP cells following PRP intervention from animal models of IVDD. A significant increase of the relative expression of ECM-related component mRNAs was observed, which included Collagen II, Aggrecan, and Sox-9, while at the same time the expression of Collagen X decreased. Additionally, TGF-β1 concentration significantly increased after PRP intervention compared to the concentration of pre-treatment levels. Specific inhibition of the TGF-β1 signaling pathway induced a significant reduction of ECM protein components and Smad2/3 expression in NP cells. The weakening of the reparative effects of PRP was caused by the inhibition of TGF-β1/Smad2/3 pathway, which led to a decrease in matrix component synthesis. These findings suggested that PRP is beneficial to NP cells and TGF-β1 plays a crucial role in the mechanism of PRP treatment for IVDD.

**Fig. 8:**
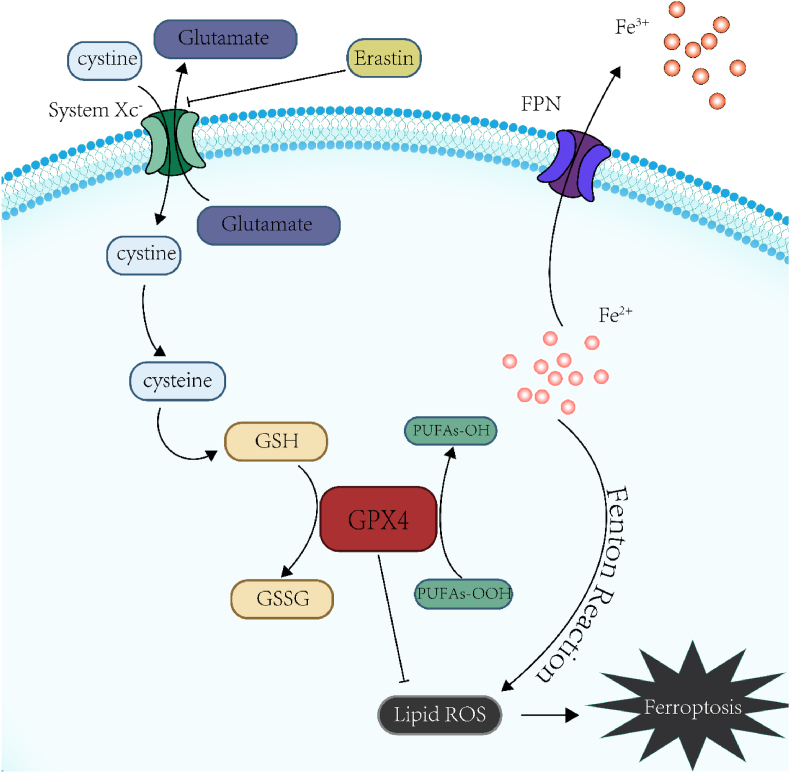
Brief schematic diagram of the mechanism of ferroptosis showing the reactions of GPX4, ROS, Fe, FPN, etc.

The biochemical characteristics of ferroptosis (Fig. 8) include the inhibition of System Xc^−^ on the cell membrane, resulting in a decrease of intracellular GSH content, GPX4 content and activity, and the weakening of inhibition function of lipid peroxidation. It will cause the increase of intracellular Fe^2+^ and ROS content simultaneously, leading to the accumulation of polyunsaturated fatty acids (PUFAs). When the intracellular Fe content increases abnormally, the Fenton reaction will cause the overload of intracellular ROS, which will trigger lipid peroxidation reaction, and the PUFAs content will increase, resulting in ferroptosis. PUFAs peroxide is produced in response to intracellular lipoxygenase, which plays a key role in ferroptosis [31,32]. System Xc-, composed of SLC7A11 and SLC3A2 [33], is an antiporter of cystine/glutamate. It is capable of transporting cystine into cells, where cystine undergoes various reactions to synthesize GSH with glutamate and glycine. GSH is critical to intracellular redox balance. The decrease in GSH content or activity of GPX4 can result in accumulation of lipid peroxidation products.

Currently, different reports have indicated that cellular Fe overload or excessive Fe accumulation can lead to the onset or progression of IVDD. Moreover, these conditions were confirmed to be closely associated with diseases related to IVDD. The connection between ferroptosis caused by intracellular iron accumulation and IVDD or IVDD related diseases has been confirmed by various studies. By establishing a mouse model of iron overload, Wang [34] et al. discovered a dose-dependent relationship among iron levels, the occurrence of IVDD and cartilage endplate degeneration. They confirmed that iron overload is an independent risk factor for human IVDD. Using ferric ammonium citrate (FAC), iron chelators, antioxidants, and ferroptosis inhibitors, they demonstrated that iron overload can lead to the degeneration of cartilage endplate cells, which provided the evidence for the correlation among ferroptosis, oxidative stress, and IVDD. Zhang [35] et al. utilized single-cell RNA sequencing and revealed the crucial role of ferroptosis-related signaling pathways in NP cells in IVDD. They also discovered that ferroptosis may represent a novel target for IVDD treatment. Xiang [36] et al. identified critical ferroptosis-related differentially expressed genes (FRDEGs) through functional enrichment analysis and protein-protein interaction network analysis (PPI). Eventually, they identified 80 FRDEGs, which were primarily associated with ferroptosis, tumor necrosis factor (TNF) signaling pathways, hypoxia-inducible factor 1 (HIF-1) signaling pathways, NOD-like receptor signaling pathways, and IL-17 signaling pathways. These findings provided new strategies for the treatment of IVDD. Sheng [37] et al. conducted a comparative analysis of chondrocytes sample from IVDD patients and control group patients. They discovered that the abnormal expression of IL-6 and IL-6R may play a crucial role in the progression of IVDD. IL-6R could downregulate miR-10a-5p levels, resulting in reduced expression of IL-6R. Furthermore, exposure to IL-6 could induce cell oxidative stress, and the disruption of iron metabolism induced to ferroptosis in chondrocyte, ultimately exacerbating the severity of IVDD. Zhang [38] et al. employed gene chip technology to perform an intersection analysis of IVDD related differential genes and ferroptosis-related genes (FRGs). They identified seven critical ferroptosis-related differentially expressed genes, including two upregulated genes, NOX4 and PIR, and five downregulated genes, TIMM9, ATF3, ENPP2, FADS2, and TFAP2A. Through this discovery, a connection between ferroptosis and immune infiltration in IVDD was established, confirming novel potential therapeutic targets for IVDD treatment. Some researchers have also explored the concept of using ferroptosis as a target for innovative IVDD treatments. Yang [39] et al. discovered that polydopamine nanoparticles (PDANPs) can inhibit ferroptosis caused by oxidative stress in NP cells. PDANPs have the capability to eliminate ROS, chelate ferrous ions, co-localize with GPX4 around mitochondria, and inhibit ubiquitin-mediated degradation reactions. PDANPs can also reduce malondialdehyde and lipid peroxides (LPO). Meanwhile, they can mitigate degeneration in the puncture induced IVDD model by suppressing ferroptosis and inhibiting the ubiquitination degradation of GPX. The studies above collectively indicated that ferroptosis plays a role in IVDD, and the ferroptosis-related mechanisms can be considered as a new therapeutic target to address the occurrence and development of IVDD.

The use of PRP or platelet-related products has also been reported in the treatment of ferroptosis-related diseases. Li [40] found that integrin β3 (ITGB3) in platelet-derived extracellular vesicles (EVs) can upregulate the expression of SLC7A11, a component of SystemXc-, by enhancing protein stability and activating the MAPK/ERK/ATF4/Nrf2 axis. SLC7A11 can promote the transfer of cystine into the interior of cells, increase the content of GSH, and thus inhibit ferroptosis. Gouel [41] and his team found that human platelet lysates (HPLs) exhibited significant neuroprotective abilities against the Parkinson's model and the amyotrophic lateral sclerosis model. HPLs are protective against apoptosis and ferroptosis, and are also able to inhibit specific oxidative stress inducers such as 1-methyl-4-phenylpyridine (MMP) and menaquinone. From the study, the effect of HPLs was more significant than that of recombinant growth factors (rGFs). Gouel also mentioned that this protective effect may be based on the MEK pathway. In the study of Mao [42], the researcher observed the effect of PRP on endometrial proliferation, and found that PRP had significant effects on the endometrial lumen, columnar epithelial morphology, number of glands, endometrial thickness, and collagen deposition. In addition, sequencing showed that the retinol metabolism pathway and the ECM receptor interaction pathway were upregulated, and they were rich in differential expression genes (DEGs), among which melanotransferrin (MELTF) was an important gene for PRP to promote endometrial proliferation. In PRP-treated Ishikawa (endometrial cancer cells) cells, ferroptosis, autophagy, pyroptosis, and other pathways were down-regulated. This also suggested that PRP can exert a therapeutic effect by modulating ferroptosis.

From the studies above, we can see the role of PRP in IVDD and the correlation between IVDD and Ferroptosis. We then deduced in this study that PRP may alleviate IVDD by ameliorating the mechanisms associated with ferroptosis. Our study suggests that PRP intervention significantly increased the levels of GPX4 and GSH-PX while reducing ROS and iron content, which may indicate that PRP inhibits ferroptosis by enhancing the antioxidant capacity of cells and reducing iron accumulation. Additionally, PRP may modulate related signaling pathways, such as TGF-β1 and PDGF-BB, to counteract the process of ferroptosis. Despite confirming the potential effects of PRP in inhibiting ferroptosis, this study has several limitations. The study was conducted solely in a rat model, and the applicability of these results to other species remains to be verified. Additionally, certain confounding factors, such as slight variations in cell culture conditions, may have influenced the results. Future research should consider increasing the sample size and validating these findings in other animal models to strengthen the conclusions drawn from this study.

## 5 Conclusions

In this study, PRP preparation and NP cells isolation and culture were successfully managed before the ferroptosis model establishment. By using PRP intervention as a key variate, we confirmed that ferroptotic state of NP cells can be improved by PRP. The results showed that PRP intervention is beneficial for ferroptotic NP cells. Our study presented a novel theoretical basis for PPR treatment of IVDD.

## CRediT authorship contribution statement

**Shi-lin Lian:** Writing – review & editing, Writing – original draft, Validation, Methodology, Formal analysis, Data curation, Conceptualization. **Jie Huang:** Writing – review & editing, Software, Methodology, Investigation, Formal analysis. **Yan Zhang:** Validation, Resources, Methodology, Investigation. **Yu Ding:** Writing – review & editing, Validation, Resources, Project administration, Funding acquisition, Data curation, Conceptualization.

## Funding

The study was supported by the Sixth Medical Center of PLA General Hospital and the National Natural Science Foundation of China (Grant No. 82274637).

## Declaration of competing interest

The authors declare that they have no known competing financial interests or personal relationships that could have appeared to influence the work reported in this paper.

## Data Availability

Data will be made available on request.
